# Interspecific Contagious Yawning in Humans

**DOI:** 10.3390/ani12151908

**Published:** 2022-07-27

**Authors:** Andrew C. Gallup, Sabina Wozny

**Affiliations:** Psychology and Evolutionary Behavioral Sciences Programs, SUNY Polytechnic Institute, Utica, NY 13502, USA; woznys@sunypoly.edu

**Keywords:** biobehavioral synchrony, circadian rhythms, human–animal interaction, empathy, stimulus detection

## Abstract

**Simple Summary:**

Contagious yawning has been observed in humans and a growing number of social vertebrates. While the majority of studies on yawn contagion have documented this phenomenon amongst conspecifics, there is also evidence for interspecific contagious yawning among non-human animals in captivity. This study was the first to formally investigate whether humans also yawn in response to yawns from different species. In particular, participants were exposed to yawning stimuli either from (1) fish, (2) amphibians, (3) reptiles, (4) birds, (5) non-primate mammals, (6) apes, or (7) domesticated cats and dogs. Overall, the results provide strong support for interspecific contagious yawning in humans, with 69% reporting yawn contagion during testing. This response was not altered by phylogenetic proximity or domestication, suggesting that the mechanisms governing yawn contagion are generalized, and can be triggered by varied representations of yawning across diverse taxa.

**Abstract:**

Contagious yawning, or the reflexive tendency to yawn following the detection of yawning in others, is well-documented among humans and a growing number of social vertebrates. While the most common form of yawn contagion occurs between conspecifics, some non-human animals in captivity have been reported to yawn in response to yawns from human handlers/caregivers. The current research sought to provide the first formal investigation of whether people yawn contagiously in response to yawns from non-human animals. In addition, this study aimed to test whether this response was modulated by phylogenetic relatedness and domestication/social closeness. A total of 296 participants from Amazon Mechanical Turk self-reported on their yawning behavior following exposure to a (1) control (non-yawning) condition or a compilation of yawning stimuli either from (2) fish, (3) amphibians, (4) reptiles, (5) birds, (6) non-primate mammals, (7) apes, or (8) domesticated cats and dogs. The results provide strong support for interspecific yawn contagion. However, neither the propensity to yawn (binary) nor total yawn frequency varied significantly across interspecific conditions. Overall, these findings suggest that the mechanisms governing yawn contagion can be activated by varied forms of yawning stimuli, including those from distantly related and unfamiliar species.

## 1. Introduction

Yawning is a complex reflex that has been documented across all classes of vertebrates [1,2,3,4]. From an evolutionary perspective, this stereotyped motor action patten appears to be a neurological adaptation that stimulates changes in state [5] and arousal [6] through intracranial circulation and brain cooling [7,8,9,10]. While yawning occurs with greatest frequency around sleeping and waking transitions [11,12,13,14], this response is also considered a displacement behavior that can be indicative of stress or conflict [15,16]. Built atop these primitive functions, yawning has taken on derived social features [17,18]. In particular, the reflexive tendency to yawn following the detection of yawns in others, i.e., contagious yawning, is a well-documented phenomenon that may serve to enhance vigilance and synchronization in groups [19]. Distinct from physiologically triggered yawns, which are ubiquitous in vertebrates, there is a great deal of variation across species when it comes to the tendency to yawn contagiously [20,21,22,23].

Psychological experiments on humans have consistently found that people yawn in response to seeing, hearing, and even thinking about other people yawning [5,24,25], while individual differences in this response are related to variability in biobehavioral synchrony [26,27]. Yawn contagion is also common among non-human great apes, including chimpanzees [28,29], bonobos [30,31], and orangutans [32]. However, studies on gorillas consistently show no evidence for this effect [33,34]. One experiment also indicated contagious yawning among a subline of high-frequency-yawning rats [35]. Observational studies of the naturalistic frequency of yawning also suggest contagion among gelada baboons [36,37], wolves [38], domesticated pigs [39], and African lions [17]. In addition, evidence for yawn contagion has been reported among African elephants [40], domesticated sheep [41], and elephant seals [42]. Outside of mammalian species, yawn contagion has also been documented in birds. In particular, budgerigars have been shown to yawn in response to both live demonstrators and visually recorded conspecifics [43,44]. However, a recent study found no evidence for contagious yawning among juvenile ravens [23].

Interspecific yawn contagion, whereby a yawn from one species elicits contagion in another species, has also been documented among some animals in captivity. In particular, chimpanzees have been shown to yawn in response to yawns from humans [45]. In one study, it was found that chimpanzees yawned contagiously both in response to yawns from humans and in-group chimpanzees, but not to out-group chimpanzees or gelada baboons, which has been interpreted as a sign of empathy [46]. However, other great apes fail to show this type of human-initiated yawn contagion, despite evidence for an intraspecific effect [33]. Chimpanzees have also been shown to yawn in response to yawns of computer animations of conspecifics [29], while orangutans do not show this reaction [32]. A recent paper also found evidence for interspecific yawn contagion among red-capped mangabeys, whereby individuals yawned more in response to conspecifics and familiar human caretakers compared to unfamiliar species (i.e., hamadryas) [47]. 

Domesticated dogs have also been reported to yawn contagiously in response to human yawns [48], which is noteworthy given that domesticated dogs fail to show intraspecific yawn contagion [49]. This discrepancy in the stimulus trigger for this response in dogs could be a result of an emphasis placed on attending to human social cues during domestication and selective breeding. A number of follow-up studies have replicated the presence of interspecific contagious yawning among dogs, with some evidence suggesting that empathy, as measured by the degree of familiarity and/or social closeness to the human yawner, enhances this response [50,51]. However, not all studies on domesticated dogs have demonstrated this type of social effect [52,53]. 

African elephants have also been shown to yawn contagiously in response to yawns from humans [54]. In particular, this study found that three out of seven captive elephants yawned following live yawns from familiar human handlers. Again, the authors propose that this behavior reflects a form of empathic processing [54]. It is important to note, however, that the connection between contagious yawning and empathy is far from clear [55], and attention towards the yawning stimuli—in this case the familiar human model(s)—could be driving this response [56].

### Current Study

To date, there have been no studies examining interspecific contagious yawning in humans. Therefore, this study sought to provide the first formal investigation of whether people yawn in response to yawns from non-human animals. Given that this phenomenon occurs in other species, and contagious yawning has proven to be a reliable phenomenon in psychological research [5,24,26,57], we predicted that humans would also show interspecific yawn contagion when compared to a control condition. Moreover, this study aimed to test whether this response was influenced by phylogenetic relatedness and domestication/social closeness. Consequently, comparisons were made between the occurrence and frequency of contagious yawning between participants that were shown yawning stimuli from the following taxonomic groupings of animals: fish, amphibians, reptiles, birds, and non-primate mammals. To more closely examine the factors of phylogeny and domestication/social closeness, yawn contagion was also measured from participants that were displayed images of yawns from apes—the closest living relatives to humans—and common household pets: domesticated dogs and cats. 

Previous studies have shown that humans have a biased perception of other animals based on phylogenic relatedness, which may be predictive of yawn contagion. For example, people tend to demonstrate both higher subjective self-report and psychophysiological measures of empathy towards species with greater phylogenetic proximity to humans [58]. Research has also shown that the perceived communicative and empathic ability of a given animal is positively correlated with the phylogenetic relatedness to humans [59]. Similarly, in a large sample of participants in the United States, Callahan et al. [60] revealed that mammals were ranked highest among traits characterized as cognitive and emotive, followed by birds, reptiles, amphibians, and then fish. Based on this literature, and the proposed connections between yawn contagion and empathy or emotional contagion [61,62], we predicted that interspecific contagious yawning in humans would be higher in response to species that were more closely related (apes > non-primate mammals > reptiles and birds > amphibians > fish). 

In addition, similar to some studies of non-human animals [63], familiarity biases for yawn contagion have previously been demonstrated in humans. In particular, observational studies report that people are more likely to yawn in response to the yawns of kin and friends compared to acquaintances and strangers [64,65]. Whether the variation in this response is driven by social/emotional closeness or enhanced attention towards people we know and care about remains unclear [56], but the effect is robust. Likewise, many of the studies on interspecific contagious yawning in captive non-human animals have been linked to some degree with empathy and/or social closeness to human owners and handlers [46,47,50,51]. Therefore, since people tend to form strong bonds and attachments with their pet cats and dogs [66,67,68], we also predicted that contagious yawning would be high in the pet condition. 

Lastly, given that physiological variables known to alter spontaneous yawning also modulate yawn contagion [27], we also hypothesized that participant tiredness at the time of testing would predict interspecies contagious yawning. Relatedly, based on the association between yawning and sleep/wake cycles [7,8,9,10], we also took into account the duration of the sleep the night prior to testing. Lastly, participant age and gender were also collected since some studies have shown these variables can affect intraspecific yawn contagion [69,70,71].

## 2. Materials and Methods

### 2.1. Participants

Participants were recruited online from Amazon Mechanical Turk (MTurk) (https://www.mturk.com/; accessed on 18 July 2022). This study was conducted in accordance with human ethics guidelines and approved by the Institutional Review Board at SUNY Polytechnic Institute (IRB-2022-3), and all participants provided informed consent prior to partaking in the study. Eligible MTurk workers were required to claim residency status in the United States, have a successful completion rate >95%, and have completed a minimum of 50 tasks. While MTurk respondents provide highly reliable data, they are less likely to pay attention to experimental materials [72]. Therefore, we recruited a relatively large sample (N = 60) per condition and implemented attention checks to improve data quality [72,73]. To screen for inattentive respondents and bots [74], we included an initial attention check question and a Completely Automated Public Turing test to tell computers and humans apart (CAPTCHA) within the demographics portion of the survey. Incorrect responses to these items excluded 32 participants. Since the effectiveness of contagious yawning stimuli is contingent upon attention towards the yawning stimuli [26,56], twelve additional attention check questions were embedded as part of the evaluation process of the contagious yawning stimuli (see design and procedure below). The presence of incorrect responses to these questions excluded another 132 participants. Finally, an additional 12 participants failed to complete the entire study after it was launched and were, therefore, excluded from the analysis. This left a total of 304 participants (167 men, 137 women; age M ± SD: 35.5 ± 10.6). 

### 2.2. Design

Online data collection was completed in Google Forms. A total of 24 images were obtained from online image searches (e.g., Google) for each of the eight conditions: control, fish, amphibians, reptiles, birds, non-primate mammals, apes, and pets. For each of the interspecific yawn conditions, twelve of these images displayed animals in mid-yawn, while the other twelve images depicted animals of the same or closely related species not yawning. Various saltwater (9) and freshwater (3) fish were represented, while frogs (8) and salamanders (4) were used for the amphibian stimuli. Reptiles included monitors (4), tortoises (2), geckos (2), iguanas (2), a chameleon (1), and a bearded dragon (1). Bird species included different wading/shorebirds (5) and birds of prey (4), a northern cardinal (1), a raven (1), and a hornbill (1). Twelve different non-primate mammals were represented, including an African elephant, camel, panda, zebra, seal, fox, koala, red panda, meerkat, rabbit, squirrel, and a laboratory rat. Apes included chimpanzees (3), gorillas (4), orangutans (3), and gibbons (2). Pets included an equal number (6) of domesticated dogs and domesticated cats. For the control condition, building windows were used as a non-yawning stimulus. In particular, twelve images of open building windows were displayed, while the other twelve images depicted similar windows that were closed. All images across conditions were standardized to 7.62 cm in height, while maintaining the original aspect ratio. Comparable yawning and non-yawning animal or open and closed window images were then paired side-by-side in randomized right/left positioning (note: all stimulus materials are available upon request from the corresponding author).

### 2.3. Procedure

Using a between-subjects design, participants were each assigned to one of the eight stimulus conditions. After providing basic demographic information (gender and age), participants were given the following instructions when viewing the contagious yawning stimuli for the condition they were assigned: “You are going to be presented with twelve pairs of images, one at a time, each depicting one animal that is yawning and one animal that is not. You need to review each image pairing and correctly identify the animal that is yawning by indicating whether it is the image on the left or the right. It is important to answer each question accurately.” Note: all references to yawning and non-yawning animals were replaced with open and closed windows in the instructions for the control condition. Since yawning (or the openness of the windows) was a salient feature to the images, incorrect responses to these questions depicted a lack of attention to the stimuli.

After viewing and responding to all twelve pairings, participants were presented with a collage of all of the yawning (or open window) images and asked to identify which appeared to be the most satisfying/appealing. This final question was not analyzed, but was included simply to enhance contagious yawning during testing. Participants were then asked to indicate whether they yawned while reviewing the stimuli (yes/no), and if so, how many times. Next, they were asked to indicate how many hours of sleep they had the previous night and respond to how tired they were on a 10-point scale (1: not tired at all; 10: extremely tired).

### 2.4. Analysis

The complete distribution of yawning frequency revealed eight outliers, as defined by 1.5 times the interquartile range. In each case, participants reported more than 12 contagious yawns during the stimulus evaluation (which took ~1 min to complete in pretesting), or greater than 1 yawn per stimulus pairing. After excluding these participants from the analysis, the final sample was 296 (control N = 38; fish N = 32; amphibians N = 38; reptiles N = 34; birds N = 34; non-primate mammals N = 37; apes N = 44; pets N = 39). A post hoc power analysis was performed using G*Power 3.1 with a medium-sized effect [75], revealing a power of 0.977 and 0.903 to detect a main effect across conditions for the binary and frequency measures of yawning, respectively. This was considered a conservative prediction given that prior work on humans has shown familiarity and social closeness to have large effects on contagious yawning [64,65].

Generalized Linear Models (GLM) were run separately for both yawn occurrence (binomial logistic) and overall frequency (Poisson loglinear). Each GLM included yawn stimulus condition and participant gender as factors, and participant age in years, sleep the previous night in hours, and current tiredness on a 1–10 scale were entered as covariates. Bonferroni corrections were applied to all post hoc comparisons. All analyses were conducted in jamovi [76] and consisted of two-tailed tests with the alpha set to 0.05.

## 3. Results

Across the sample, 69.0% of participants reported yawning contagiously in response to the interspecific stimuli, with a median of two yawns/participant. In comparison, only 28.9% of participants reported yawning in response to the control stimuli, with a median of 0 yawns/participant. Descriptive statistics for non-yawning variables are presented in Table 1.

**Figure 1:**
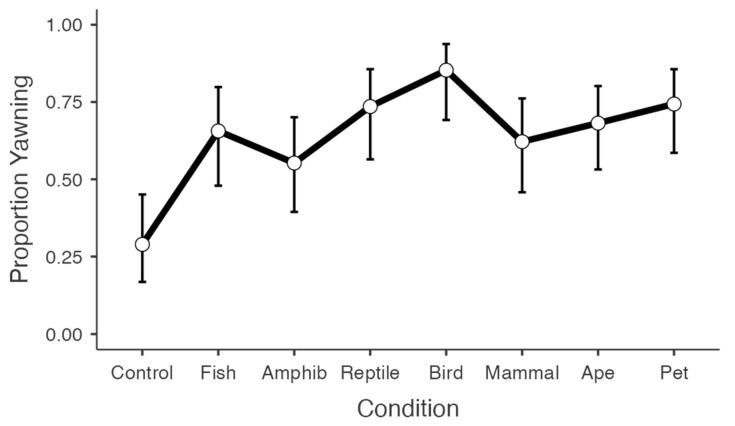
Line graph depicting the proportion of yawners and non-yawners across conditions. Note: data are presented as *M* ± 95% CI.

For the binomial measure of yawning (yes/no), both the stimulus condition and the tiredness of participants were significant predictors (Table 2). Participants in each of the interspecific yawning conditions were more likely to report yawning compared to the control condition (Figure 1). Contrary to our predictions, however, post hoc comparisons revealed that the proportion of contagious yawners did not vary across the seven interspecific yawning conditions (ps > 0.05).

**Figure 2:**
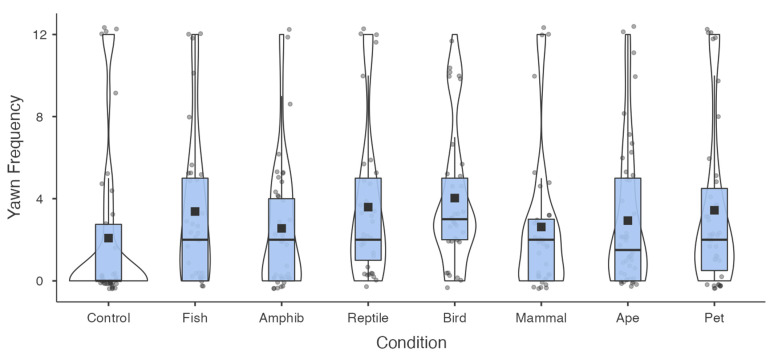
Box and violin plots depicting the frequency of contagious yawning across conditions. Box plots represent the median, interquartile ranges, and the whiskers extend 1.5 times the interquartile range for the upper and lower boundary, while the violin plots illustrate the distribution of yawn frequency. Observed responses (grey circles) and group means (black boxes) are also represented.

For the yawn frequency model, stimulus condition and the tiredness of participants were again both significant predictors (Table 3). Participants across the interspecific yawning conditions, with the exception of the amphibian and mammal conditions, reported a greater number of yawns compared to the control condition (Figure 2). Again, post hoc comparisons revealed that the frequency of contagious yawning did not vary significantly across the seven interspecific yawning conditions (*p* > 0.05).

## 4. Discussion

Contagious yawning is well-documented in both naturalistic and experimental studies on humans [5,24,57,65,77], and emerges during early childhood development [78]. While previous comparative research has provided evidence for interspecific, i.e., human-initiated, contagious yawning in chimpanzees [45,46], red-capped mangabeys [47], domesticated dogs [48,50,51,53], and African elephants [54], to date, there have been no studies examining whether humans yawn contagiously in response to non-human animals.

The current findings provide strong support for interspecific contagious yawning in humans. The tendency to yawn was significantly higher in each of the interspecific yawning conditions compared to the control (non-yawning) condition. The same was true for overall yawn frequency, except in the case of the amphibian and mammal conditions. The attentional checks to the pairing of yawn and non-yawning stimuli produced a robust response, with a comparably high rate of yawn contagion (69%) to a recent online study on intraspecific yawn contagion using compiled video clips [27]. Contrary to our predictions, however, neither phylogenetic proximity nor domestication/social closeness of the yawning stimuli enhanced this response. In fact, there was a complete absence of any trend consistent with these hypotheses (Figure 1 and Figure 2). Since previous work has shown that people tend to have higher levels of empathy towards species with greater phylogenetic proximity [58], these findings do not support the view that contagious yawning is linked with empathy or emotional contagion [46,63,64,65]. Instead, the current results suggest that the mechanisms governing yawn contagion in humans are generalized, and can be triggered by varied representations of yawning across diverse taxa. Likewise, since the inclusion criteria for this study required stringent attention checks towards the yawning stimuli, these findings are consistent with the position that variation in yawn contagion is driven by detection of the yawning stimulus [55,56].

Similar to most studies of intraspecific contagious yawning [79], participant gender was not a significant predictor of interspecific yawn contagion. However, as predicted, participants that were more tired at the time of testing reported both a higher incidence and a greater overall frequency of yawning (see Table 2 and Table 3). The fact that tiredness was the best predictor of yawn contagion in this study replicates recent research on intraspecific contagious yawning in humans [27] and further supports previous studies showing that contagious yawns are modulated by physiological factors known to trigger spontaneous yawning [8,80,81,82,83].

While this study provides novel results and an improved understanding of contagious yawning, there are limitations that should be acknowledged. First, only twelve yawning images were included in each interspecific condition, and with the exception of the apes, this only represented a small proportion of species diversity within the taxonomic groupings. Therefore, it remains possible that different species and/or representations of yawning could produce different results. However, we find this unlikely given the complete absence of any trend for phylogenetic proximity or domestication increasing contagion. Nonetheless, future research could be conducted to potentially identify species that elicit stronger or weaker responses. For example, based on this study, we could not assess whether contagion in the pet condition differed between cat and dog stimuli. The online nature of this study is another limitation, as it relied on self-reported contagious yawning from respondents using MTurk. However, previous studies with diverse methodologies have shown that self-report is a valid measure of contagious yawning [25,84,85]. In addition, the attention check questions used when evaluating the stimuli exceed typical attentional measures in studies on yawn contagion in humans and, thus, represent a strength of the current research. In particular, this method ensured that all yawning stimuli were actually detected, rather than attention being directed towards the stimulus images in general. That said, we hope this initial study spurs follow-up research in this area.

## 5. Conclusions

In summary, this research provides the first evidence for interspecific contagious yawning in humans. Contrary to our predictions, the results show that this response was not enhanced by phylogenetic proximity or domestication/social closeness. Instead, these findings suggest that, when controlling for attention, the mechanisms governing yawn contagion can be activated by varied forms of yawning stimuli, including those from distantly related and unfamiliar species.

## Figures and Tables

**Table 1:** Descriptive statistics.

	Variable			
Condition	Gender (M:F)	Age (years)	Sleep (h)	Tired (1–10)
Control	20:18	34.1 ± 10.2	7.21 ± 1.04	5.41 ± 3.26
Fish	20:12	36.4 ± 11.5	7.36 ±1.46	6.00 ± 2.90
Amphibians	21:17	37.0 ± 10.7	6.89 ± 1.64	5.18 ± 3.02
Reptiles	19:15	33.7 ± 9.1	7.24 ± 1.69	5.74 ± 3.11
Birds	20:14	34.1 ± 10.4	7.18 ± 1.51	7.21 ± 2.74
Mammals	19:18	33.2 ± 10.1	6.89 ±1.02	5.62 ± 3.23
Apes	25:19	37.7 ± 11.2	7.14 ± 1.29	5.52 ± 2.55
Pets	20:19	36.8 ± 11.5	7.06 ± 1.26	6.38 ± 2.98
Combined	164:132	35.5 ± 10.7	7.11 ± 1.37	5.83 ± 3.00

Final sample N = 296. Combined measures represent the total ratio or overall *M* ± SD. Note: F = female; M = male.

**Table 2:** Estimated parameters (Estimate), Standard Error (SE), and results of the likelihood ratio tests (χ^2^) for the binomial logit distribution.

Fixed Factors	Estimate	SE	*df*	χ^2^	*p*-Value
Intercept	0.829	0.164	*-*	*-*	*-*
Condition			7	27.368	<0.001
Fish ^a,b^	1.880	0.629	1		0.003
Amphibians ^a,b^	1.728	0.599	1		0.004
Reptiles ^a,b^	2.532	0.656	1		<0.001
Birds ^a,b^	2.767	0.730	1		<0.001
Mammals ^a,b^	1.850	0.615	1		0.003
Apes ^a,b^	2.274	0.586	1		<0.001
Pets ^a,b^	2.348	0.635	1		<0.001
Gender (Female) ^a,b^	0.151	0.319	1	0.223	0.637
Age (Years)	−0.029	0.015	1	3.666	0.056
Sleep (Hours)	0.093	0.120	1	0.590	0.442
Tiredness (1–10)	0.441	0.059	1	73.402	<0.001

^a^ Estimate ± SE refers to the difference in the response between the reported level of this categorical predictor and the reference category of the same predictor; ^b^ “Condition (Control)” and “Gender (Male)” were the reference categories.

**Table 3:** Estimated parameters (Estimate), Standard Error (SE), and results of the likelihood ratio tests (χ^2^) for the Poisson distribution.

Fixed Factors	Estimate	SE	*df*	χ^2^	*p*-Value
Intercept	0.964	0.039	-	*-*	*-*
Condition			7	14.648	0.041
Fish ^a,b^	0.375	0.149	1		0.012
Amphibians	0.247	0.153	1		0.107
Reptiles	0.481	0.144	1		<0.001
Birds	0.364	0.142	1		0.011
Mammals	0.178	0.152	1		0.242
Apes	0.343	0.144	1		0.017
Pets	0.311	0.143	1		0.030
Gender (F)	0.035	0.068	1	0.267	0.605
Age (years)	0.003	0.003	1	1.017	0.313
Sleep (h)	0.028	0.023	1	1.495	0.222
Tiredness (1–10)	0.187	0.014	1	207.637	<0.001

^a^ Estimate ± SE refers to the difference in the response between the reported level of this categorical predictor and the reference category of the same predictor; ^b^ “Condition (Control)” and “Gender (Male)” were the reference categories.

## Data Availability

The datasets used to generate the results are provided here: https://doi.org/10.7910/DVN/3STP5S (accessed on 25 July 2022).
